# A greener vision for vector control: The example of the Singapore dengue control programme

**DOI:** 10.1371/journal.pntd.0008428

**Published:** 2020-08-27

**Authors:** Shuzhen Sim, Lee Ching Ng, Steve W. Lindsay, Anne L. Wilson

**Affiliations:** 1 Environmental Health Institute, National Environment Agency, Singapore; 2 Department of Biosciences, Durham University, Durham, United Kingdom; 3 Department of Vector Biology, Liverpool School of Tropical Medicine, Liverpool, United Kingdom; Faculty of Science, Mahidol University, THAILAND

## Abstract

Vector-borne diseases are a major cause of morbidity and mortality worldwide. *Aedes*-borne diseases, in particular, including dengue, chikungunya, yellow fever, and Zika, are increasing at an alarming rate due to urbanisation, population movement, weak vector control programmes, and climate change. The World Health Organization calls for strengthening of vector control programmes in line with the Global Vector Control Response (GVCR) strategy, and many vector control programmes are transitioning to this new approach. The Singapore dengue control programme, situated within the country’s larger vision of a clean, green, and sustainable environment for the health and well-being of its citizens, provides an excellent example of the GVCR approach in action. Since establishing vector control operations in the 1960s, the Singapore dengue control programme succeeded in reducing the dengue force of infection 10-fold by the 1990s and has maintained it at low levels ever since. Key to this success is consideration of dengue as an environmental disease, with a strong focus on source reduction and other environmental management methods as the dominant vector control strategy. The programme collaborates closely with other government ministries, as well as town councils, communities, the private sector, and academic and research institutions. Community engagement programmes encourage source reduction, and house-to-house inspections accompanied by a strong legislative framework with monetary penalties help to support compliance. Strong vector and epidemiological surveillance means that routine control activities can be heightened to specifically target dengue clusters. Despite its success, the programme continues to innovate to tackle challenges such as climate change, low herd immunity, and manpower constraints. Initiatives include development of novel vector controls such as *Wolbachia*-infected males and spatiotemporal models for dengue risk assessment. Lessons learnt from the Singapore programme can be applied to other settings, even those less well-resourced than Singapore, for more effective vector control.

## Author summary

Infections due to dengue and other viruses transmitted by *Aedes* mosquitoes are on the rise worldwide as a result of urbanisation, trade, population movement, and climate change. Contemporary vector control programmes have struggled to control *Aedes*-borne diseases, but the Singapore dengue control programme provides a success story. Key to the success is consideration of dengue as an environmental disease, with a corresponding focus on removal of water containers from in and around homes, solid waste management, and limited use of insecticides. The programme relies on close collaboration with other government and nongovernment actors including communities. Community engagement campaigns to ensure high awareness are supported by house-to-house inspections for mosquito habitats and a legislative framework including penalties for noncompliance. The Singapore dengue control programme provides important lessons that can be applied to other *Aedes* control programmes and vector control programmes more widely.

## Introduction

**Fig 1:**
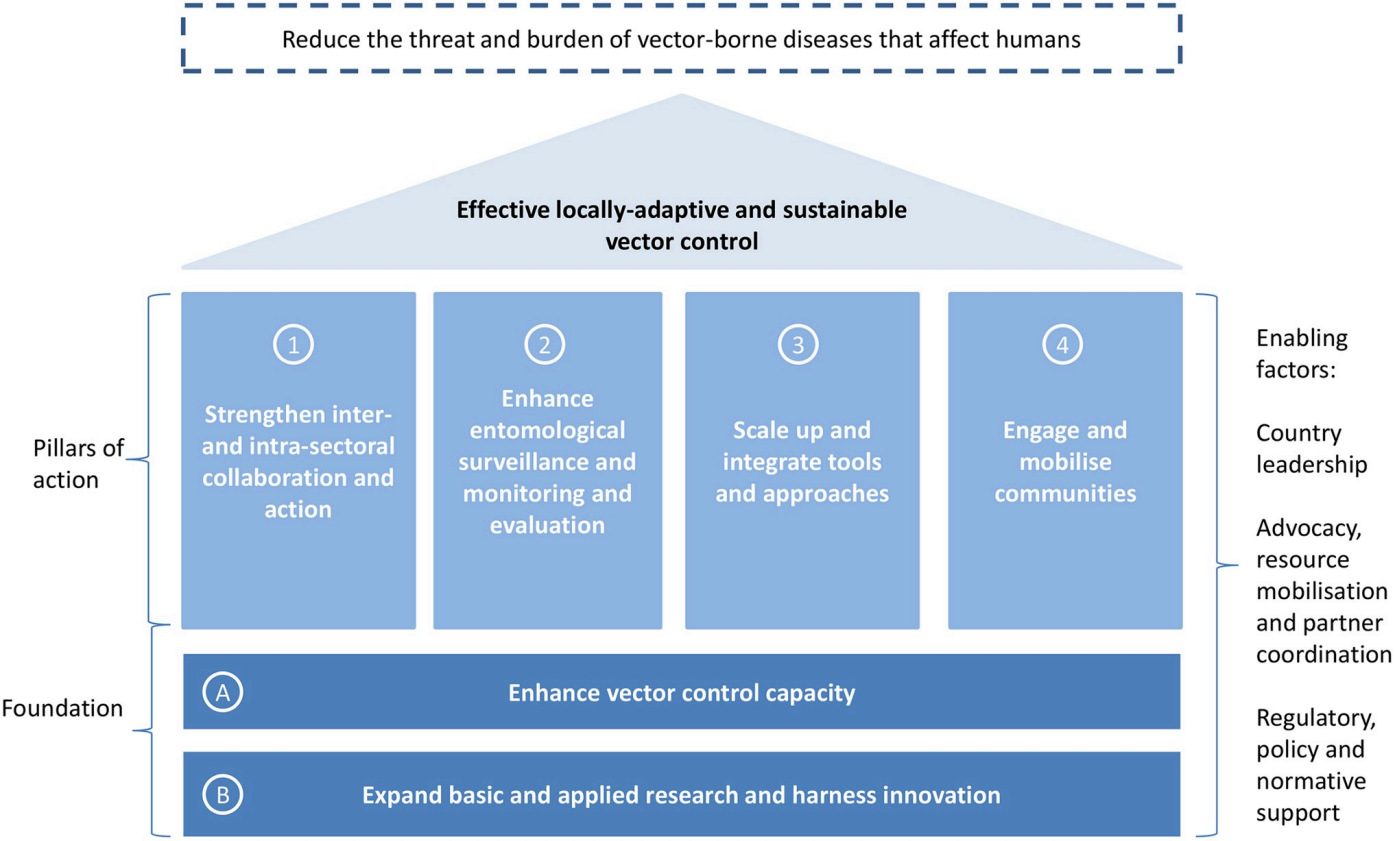
Global Vector Control Response Framework [9].

Vector-borne diseases (VBDs) are a major cause of morbidity and mortality in the tropics and subtropics, accounting for more than 17% of the global burden of infectious diseases [1], with over 80% of the world’s population at risk from at least one VBD [2]. *Aedes*-borne diseases, including dengue, chikungunya, yellow fever, and Zika, are increasing at an alarming rate driven by urbanisation, local and global population movement, and climate change [3–5]. *Aedes aegypti* mosquitoes lay eggs in a wide variety of artificial containers and structures, including those that occur in discarded plastic containers, water storage containers, flowerpots, tyres, poorly constructed concrete structures in the ground, and gutters, which abound in urban environments. The main tool we have for controlling VBDs is vector control [6]. Although vector control has been hugely successful against some VBDs such as malaria [7], weak implementation of sustainable programmatic vector control has struggled to control *Aedes*-borne diseases in many parts of the world in the past 20 years. Margaret Chan, former Director-General of the World Health Organization (WHO), famously said in her 2016 World Health Assembly address that the Zika epidemic was "the price being paid for a massive policy failure that dropped the ball on mosquito control in the 1970s" [8]. In order to strengthen vector control, WHO member states called on WHO to develop the Global Vector Control Response 2017–2030 (GVCR) [9]. This strategic document includes a framework of key priorities for vector control strengthening. The GVCR calls for collaboration within and outside the health sector, increased engagement of communities, scaling up and integration of vector control tools and approaches, and improved surveillance and monitoring and evaluation (Fig 1). This is supported by a foundation of capacity and capability strengthening and by increased basic and applied research and innovation. Also important are factors including country leadership, advocacy, resource mobilisation, and regulatory, policy, and normative support. WHO member states are reorienting their programmes in line with the GVCR [10], but good examples of the GVCR principles in action are currently limited. One exception is the dengue control programme in Singapore, which in fact predates the GVCR but adopts many GVCR principles. This manuscript aims to document the key features of the programme, highlight how they sit within the GVCR framework, and draw out important lessons that can be applied by other vector control programmes, including those less well-resourced than Singapore’s.

### The Singapore context

The Republic of Singapore is an island city-state in Southeast Asia with a land area of 724.2 km^2^ and a population of 5.64 million [11, 12]. Singapore is located at the southern tip of the Malay peninsula, connected to Peninsular Malaysia via two causeways and by ferry services with Indonesia’s Riau islands to the south. Singapore is a travel and business hub with 18.5 million visitors in 2018 [13]. The city-state is highly urbanised and has a high population density with almost 8,000 people per km^2^, and typically tens of thousands of people per km^2^ in the main residential areas [14]. Since 1963, Singapore has been working towards the vision of creating a ‘City in a Garden’, where greenery and biodiversity are seen as solutions to improving health and well-being of its citizens [15–17]. Seventy-nine percent of Singapore’s residents live in high-rise apartments built by the government’s Housing and Development Board (HDB), with the remainder living in privately owned condominiums (16%) or landed property (5%) [18]. The climate in Singapore is tropical and it is hot and humid all year round. Singapore is endemic for dengue, with all four dengue virus (DENV) serotypes circulating, frequent emergence of various genotypes, and a cyclical pattern of outbreaks every 5–7 years [19–22]. In 2005, 2007, 2013, and 2016, Singapore experienced explosive dengue fever outbreaks that resulted in 14,032, 8,287, 22,170, and 12,848 indigenous cases, respectively, with incidence rates of 322.5, 180.6, 404.9, and 229.1 per 100,000 population [23–26]. In 2019, Singapore also experienced a large dengue outbreak with more than 16,000 cases [27]. There is low herd immunity, particularly among younger generations, due to decades of low dengue transmission [28–30]. The primary dengue vector is *A*. *aegypti*, and although *Aedes albopictus* is also present, it is considered a weak secondary vector [31]. Exposure to chikungunya is low in Singapore, with seroprevalence of 1.9% among adults [32]. The country has experienced small local outbreaks of chikungunya, with the largest (1,011 cases and an incidence rate of 18.7 per 100,000) occurring in 2013, a year when *Aedes* densities were high [25]. Singapore’s first Zika outbreak occurred in 2016, and although it occurred at the same time as the epidemic in Brazil and other Latin American countries, the Zika strain was found to have originated in Southeast Asia [33]. Malaria was eliminated in Singapore in 1981 [34], but there are still sporadic imported cases and competent vectors including *Anopheles epiroticus* (formally *Anopheles sundaicus*), *Anopheles maculatus*, and the emerging vector *Anopheles sinensis* [35, 36].

### Singapore’s greener vision

Since independence, Singapore has recognised that a clean and green environment is necessary not only to ensure good health and a good quality of life for its people but also for economic competitiveness. The government embarked on projects to clean up the land and waterways and invested heavily in critical sanitation and environmental infrastructure, including drainage development projects, sewerage and used water treatment infrastructure, and solid waste management. The environmental management put in place to implement this high standard of public cleanliness has greatly benefited Singapore’s efforts to tackle VBDs. Underscoring the view that *Aedes*-borne diseases are environmental diseases, dengue control in Singapore is led by the National Environment Agency (NEA), a statutory board of the Ministry of the Environment and Water Resources (MEWR).

### Intra- and intersectoral collaboration

**Fig 2:**
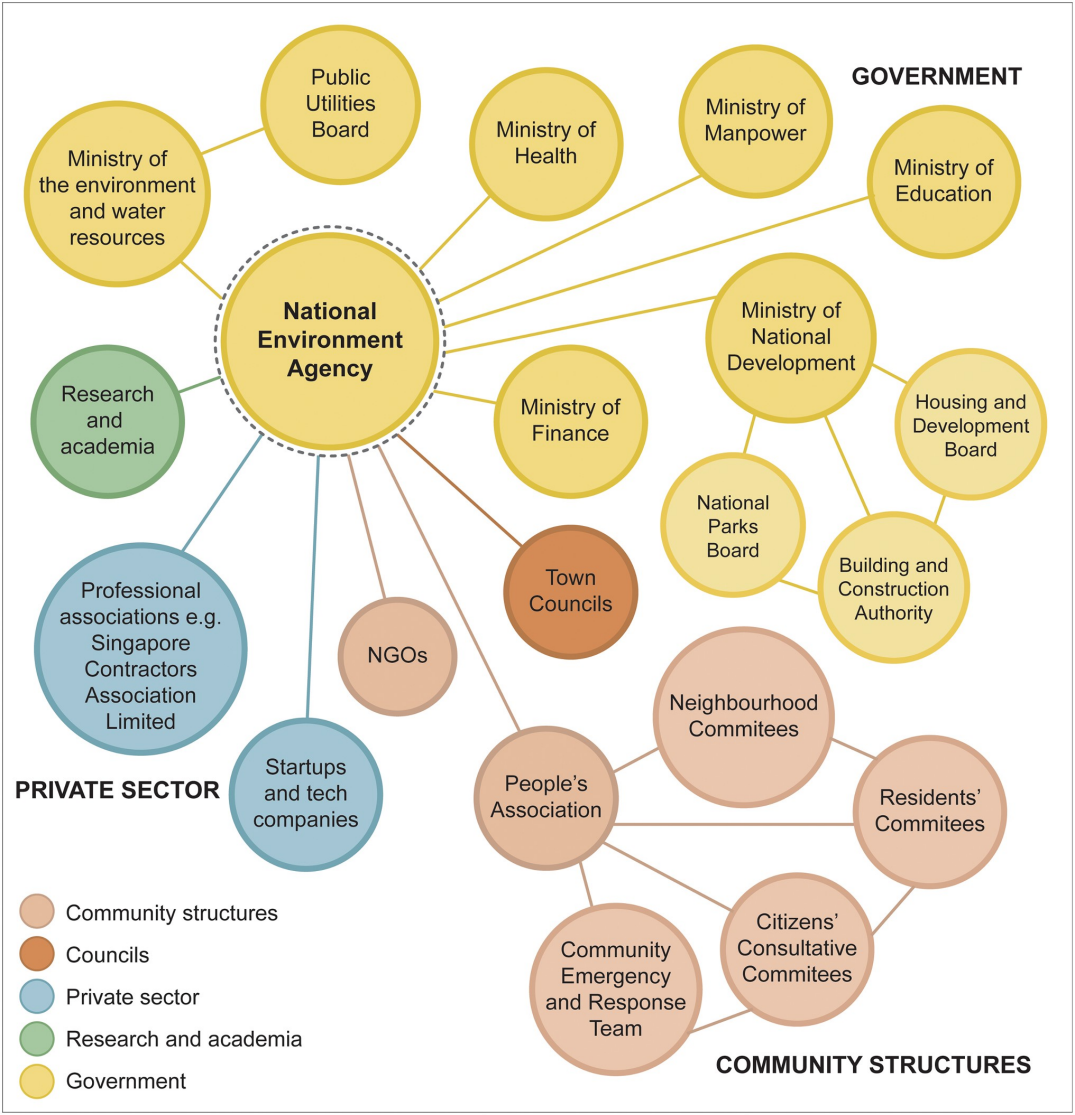
Collaboration between National Environment Agency and other sectoral stakeholders. NGO, nongovernmental organisation.

In view of the importance of infrastructure maintenance and design, environmental sanitation, people’s behaviours, and use of technologies on dengue prevention, the NEA collaborates closely with other government ministries (e.g., Health, National Development, Education, Finance), town councils (responsible for management and maintenance of the common property of public housing estates, including vector control), community associations, research and academic institutions, and the private sector (Fig 2). Intersectoral activities are coordinated by an Inter-Agency Dengue Taskforce which meets regularly, and monthly during outbreaks. In the event of a severe dengue outbreak, the Taskforce membership is escalated to Minister level. In addition to regular meetings, Taskforce members are also in close contact via email and telephone to exchange feedback and information—for example, on unusual aquatic habitats—in a timely manner.

Resources for government agencies to carry out dengue control activities are allocated by the Ministry of Finance, to which each agency justifies its own funding needs. If necessary, NEA supports these justifications, especially in an outbreak year when NEA has called on agencies to step up dengue control measures. NEA may also offer subsidies to town councils to support enhanced dengue control measures in public housing estates.

**Fig 3:**
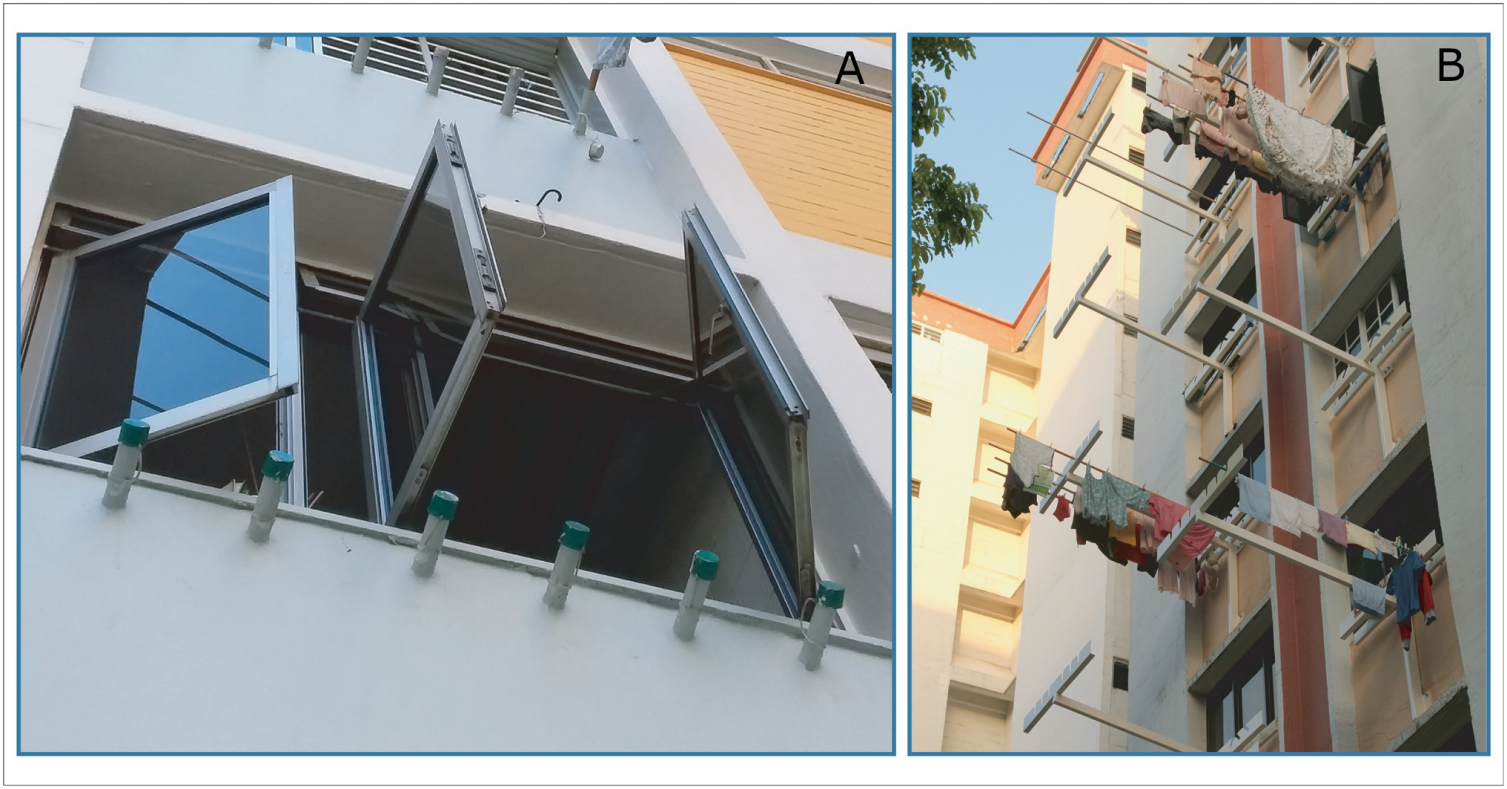
Redesign of washing line construction from bamboo pole holders with caps (A) to novel rack system (B).

For example, an important partner of the NEA is the HDB, the statutory board responsible for public housing. In high-rise apartments in Singapore, laundry is typically hung out to dry on bamboo poles fixed into tube-shaped bamboo pole holders on the side of the building. Discovery of substantial numbers of *Aedes* larvae in these holders led to provision of caps to cover them when not in use. Later, the clothes-drying system was redesigned such that the bamboo poles now rest on brackets instead, eliminating the need for holders (Fig 3). As mounting the bamboo poles in the holders required residents to lean out of the window, replacing the holders with brackets not only removed a mosquito breeding habitat but was also safer for residents. Antimosquito valves that allow water to drain away but prevent escape of mosquitoes are installed in gully drains in HDB apartments, and HDB blocks are constructed without roof gutters, since these are difficult to access and maintain at height and therefore can become *Aedes* habitats. NEA works closely with town councils who are responsible for vector control around the HDB blocks.

**Fig 4:**
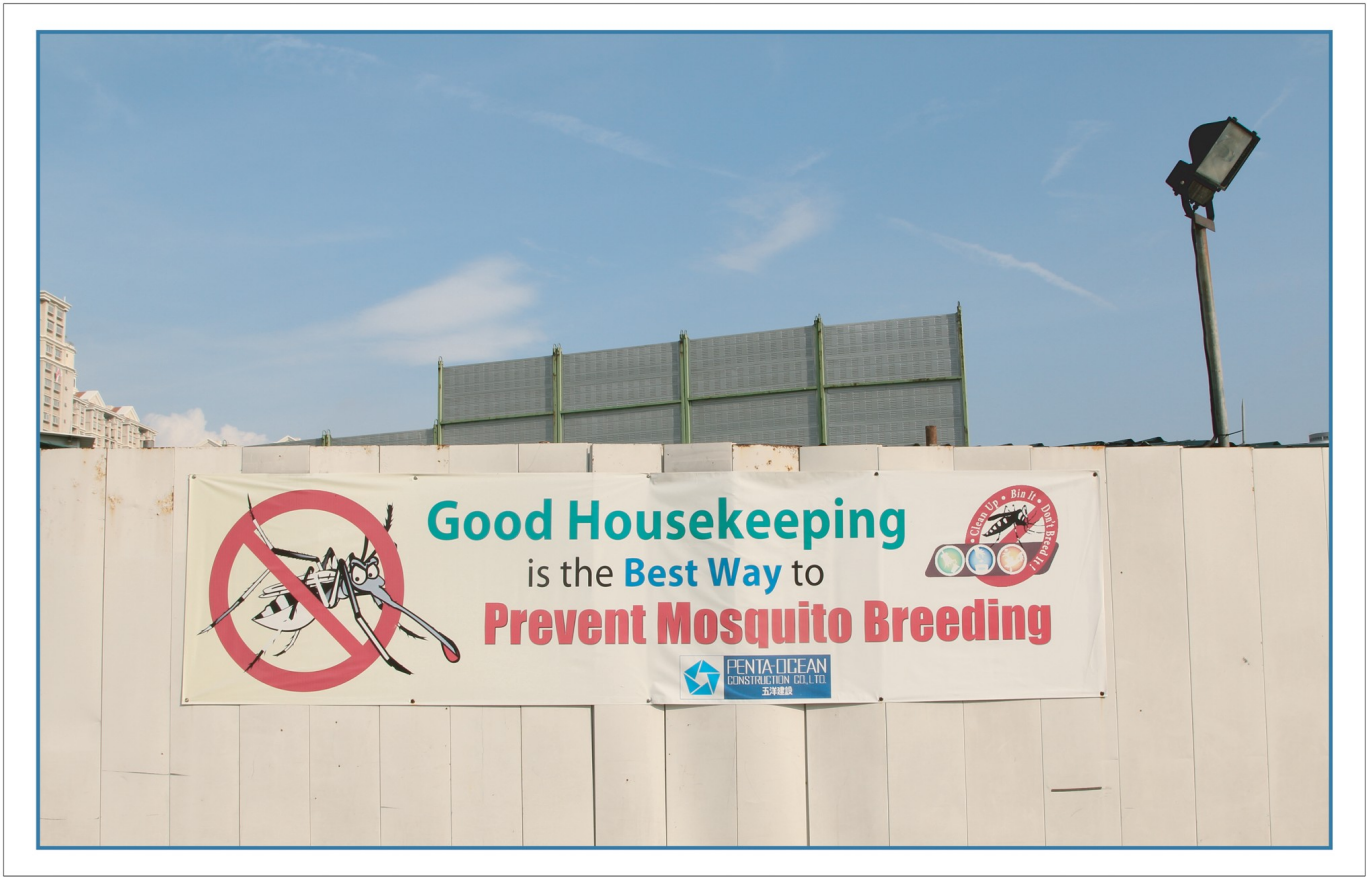
Banners at construction sites to encourage good housekeeping to reduce *Aedes* habitats.

Construction sites play an important role as a driver of sustained dengue transmission [37], since rainwater-filled land excavation holes, construction materials, and equipment (e.g., water tanks, skips, canvas sheeting) can become *Aedes* habitats. The Singapore Contractors Association Limited (SCAL) is therefore an important partner for NEA. Construction sites are mandated to engage an Environmental Control Officer (large sites may even have their own dedicated Officer) who ensures appropriate action is taken to reduce vector proliferation, including larviciding with *Bacillus thuringiensis israelensis* (*Bti*) (Fig 4). The Land Transport Authority is responsible for good housekeeping and vector control on their infrastructure construction sites and the Urban Redevelopment Authority conducts vector control along roads and their car parks.

Within the NEA, the Department of Public Cleanliness maintains a system of daily litter collection and its mandate is cleaning to ‘public health standards’. Approximately 60% of Singapore’s waste is recycled and the remainder incinerated, with the waste ash deposited on an offshore landfill site, Semakau Island, helping expand the island. This serves to reduce artificial containers in the environment which could otherwise become habitats for *Aedes* mosquitoes.

Within the MEWR, the NEA works alongside the Public Utilities Board (PUB), which is responsible for ensuring a sustainable and efficient water supply and responsible for drainage system in Singapore. Vector control activities of the PUB include designing drains for maintainability (accessible and with sufficient gradient to prevent water pooling) and regular flushing of drains monthly or bimonthly. The Department of Public Cleanliness conducts regular cleaning of drains to remove any debris or refuse.

**Fig 5:**
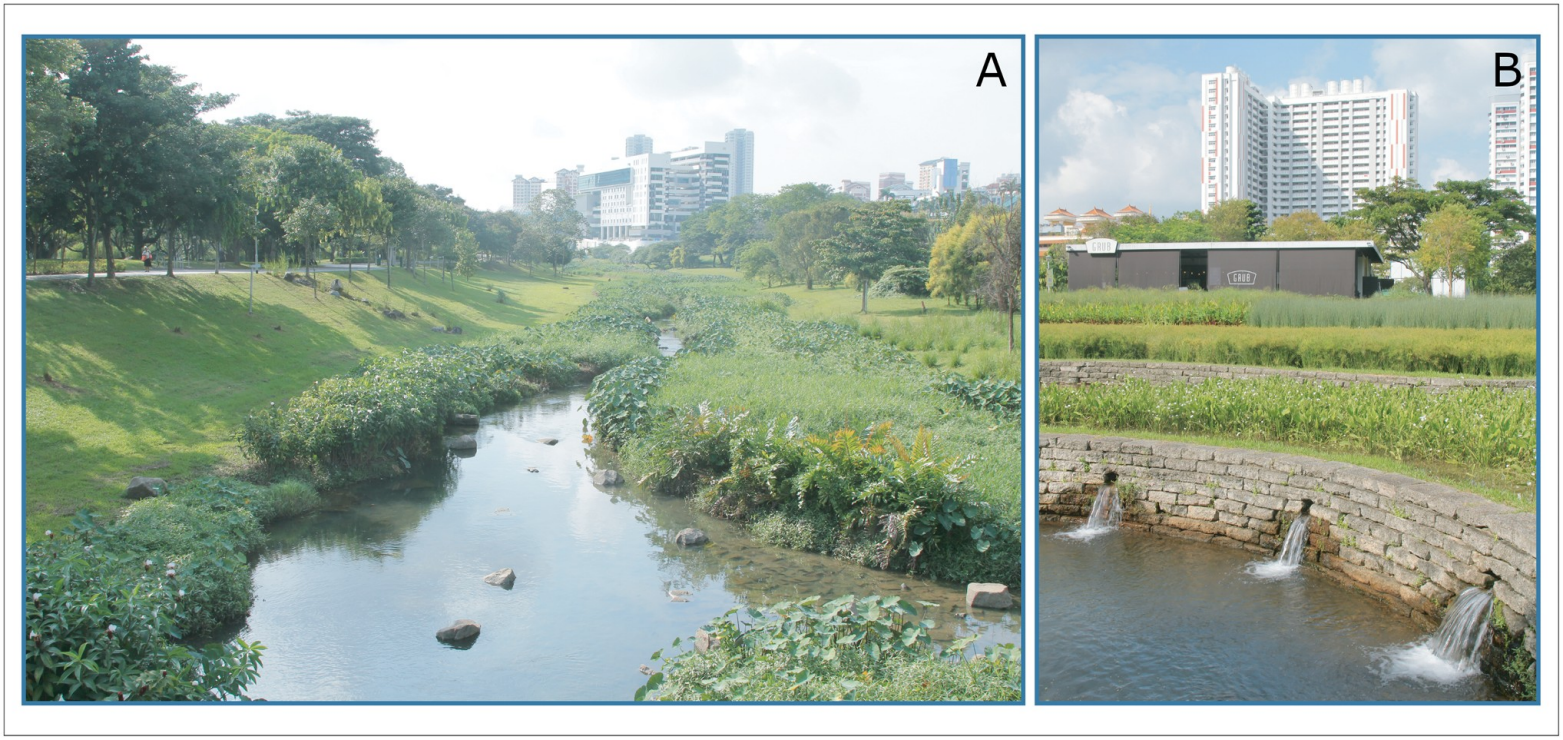
Vector control measures in Bishan-Ang Mo Kio Park. (A) Stream treated monthly with *Bti* by the Public Utilities Board. (B) Papyrus beds treated with *Bti* and dried intermittently to reduce biting midges and *A*. *sinensis*. *Bti*, *Bacillus thuringiensis israelensis*.

The National Parks Board (NParks) also works closely with NEA to maintain a pleasant environment with trees, plants, and water features such as ponds and streams, while keeping vector densities to a minimum. Vector control measures implemented include weekly *Bti* treatment of ponds and regular removal of floating vegetation. If vectors do become a problem, surveillance and vector control measures are quickly put in place. For example, in the first quarter of 2019, ornamental papyrus beds in Bishan-Ang Mo Kio Park encouraged the proliferation of biting midges and *A*. *sinensis*. NEA’s identification of the vectors responsible led to control measures including *Bti* application, intermittent drying of the papyrus beds, and human behaviour change to encourage use of topical repellent by nearby café customers in the evening (Fig 5).

### Engagement and mobilisation of communities

The NEA Corporate Communications Department is responsible for mass and social media communications, whereas the 3P (People, Private, Public) Network Division leads NEA’s education and publicity initiatives and programmes for the 3P sectors, across all environmental initiatives, not just dengue prevention. Community engagement makes use of existing structures including the People’s Association (the statutory board responsible for promoting social cohesion) and grassroots organisations under the People’s Association, such as Citizens’ Consultative Committees, Residents’ Committees in HDB estates, and Neighbourhood Committees in private housing estates. These groups encourage bottom-up participation by seeking residents’ cooperation in checking for mosquito breeding in homes. NEA also trains members of the public and the People’s Association’s Community Emergency Response Teams as Dengue Prevention Volunteers, who educate fellow residents on dengue prevention.

**Fig 6:**
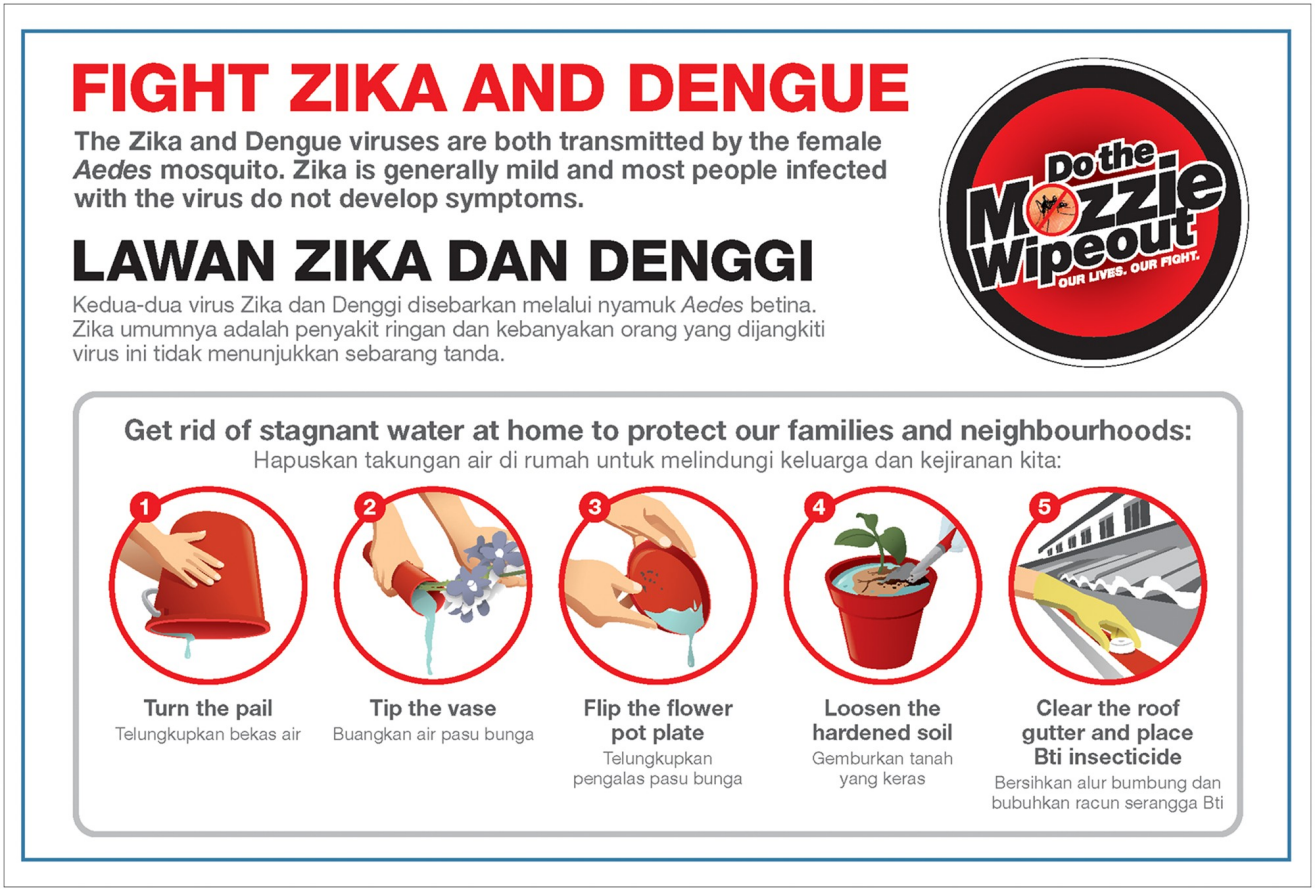
‘5-Step Mozzie Wipeout’ behaviour change communication materials.

Each year the 3P Network Division organises the National Dengue Prevention Campaign, with the timing of the launch dependent on the dengue forecast provided by the Environmental Health Institute (EHI), the research arm of NEA. 3P work through the mayors of each district, grassroots members, and Dengue Prevention Volunteers who mobilise their communities to conduct source reduction according to the ‘5-Step Mozzie Wipeout’ by doing house-to-house visits, distributing educational materials, and organising block parties and other events. The ‘5-Step Mozzie Wipeout’ targets the top five most common habitats in residential premises in Singapore. Information materials are available in all four written national languages (Fig 6). The ‘5-Step Mozzie Wipeout’ recommends to (1) turn the pail, (2) tip the vase, (3) flip the flowerpot plate, (4) loosen the hardened soil, and (5) clear the roof gutter and place *Bti* larvicide inside. For those interested in *Aedes* control outside Singapore, it is important to note that these aquatic habitats may not be the top five habitats in other countries, so it is important to survey local sites to identify the major sources of *A*. *aegypti*. In Singapore, messaging is directed towards people taking personal responsibility for mosquito prevention and explaining that by conducting source reduction, they can protect their families from dengue. 3P uses different methods to evaluate knowledge, attitudes, and practices of communities over time and looks for fresh angles to prevent fatigue from regular public health messaging.

Besides nationwide general messaging, community engagement strategies also target specific population groups to encourage them to play a greater role in mosquito prevention, including domestic helpers, construction workers, the elderly, and school children. For example, domestic helpers and construction workers are targeted with behaviour change messaging through outreach and roadshows at dormitories, shopping malls, and other places of congregation. Since these groups are often migrants and therefore transient populations, behaviour change materials are produced in the relevant languages (e.g., Bahasa, Hindi, Indonesian, Tagalog) and outreach is conducted regularly. Dengue prevention roadshows are also conducted at Elderly Care Corners and videos are available in local Chinese dialects used by many elderly residents who originate from mainland China. The 3P Network Division partners with the Ministry of Education to include dengue prevention in school curricula at primary, secondary, and tertiary levels.

**Fig 7:**
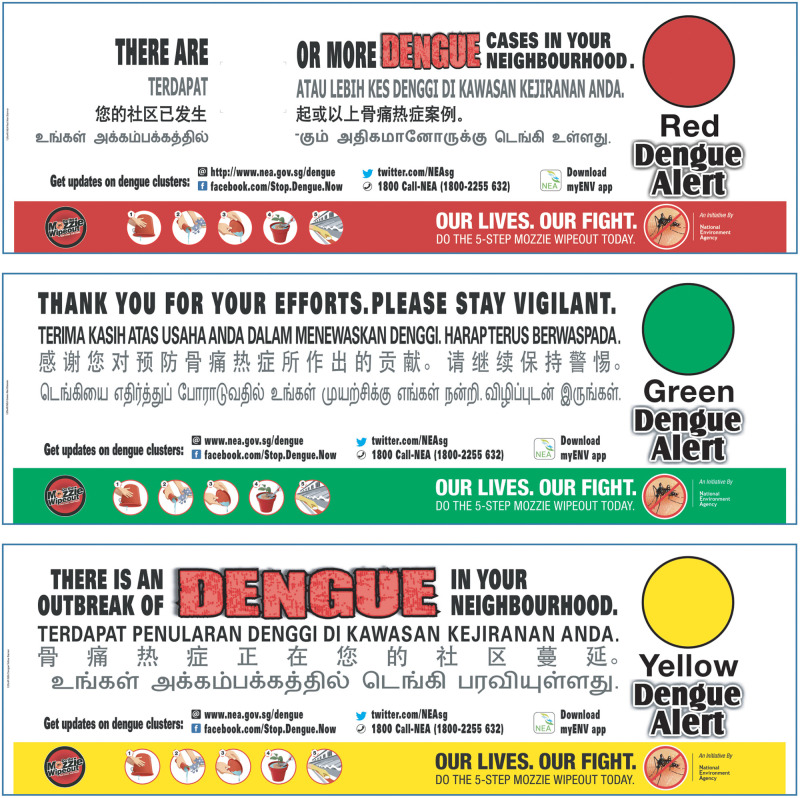
Colour-coded community dengue alert system.

Timely information on the number and location of dengue cases is made publicly available on the NEA website and MyENV mobile app. NEA also implements a Community Dengue Alert System, in the form of colour-coded banners placed in high-visibility locations in dengue clusters to inform residents about the dengue situation in their neighbourhood and the corresponding actions they can take (Fig 7). The banners use the widely recognisable ‘traffic light’ colour code of red (high alert), yellow (medium alert), and green (low alert) to allow for easy interpretation of the situation. Communities are engaged and regularly give feedback about, for example, increased mosquito numbers, littering, and other environmental issues either online or via a 24-hour NEA hotline.

### Vector surveillance and monitoring and evaluation of interventions

Globally, *Aedes* indices such as the house index (HI, percentage of houses positive for larvae and or pupae) are traditionally used for *Aedes* vector monitoring, with a value of 1% often being used as an arbitrary dengue transmission threshold [38, 39]. The Singapore dengue control programme has brought the HI to very low levels (from 50% in the 1960s to 0.3% in the 2000s). Despite this low HI, Singapore still experiences regular outbreaks, suggesting that the HI is no longer sensitive for dengue risk assessment [40, 41]. With low vector populations, the Singapore programme now uses in-house–developed Gravitraps, which are designed to lure and trap gravid female *Aedes* on the sticky lining [31]. Since 2017, a system of 50,000 Gravitraps have been deployed in public housing estates nationwide, and the scheme will be expanded to landed housing by end of 2019 [31]. Monitoring of the traps every 2 weeks is currently outsourced, although an automated trap is currently under development by NEA. Gravitrap indices are plotted on a geographical information system (GIS) and used to target intensified source-reduction campaigns. Areas with a high Gravitrap index are also published on the NEA website to motivate the local community to take action to reduce dengue transmission. In keeping with the country’s green aspirations, insecticides are used judiciously, with *Bti* and temephos used as larvicides and pirimiphos-methyl as adulticide. Choice of insecticide is guided by resistance monitoring, which is performed every few years. As well as *Aedes*, surveillance is also conducted for culicines (potential Japanese encephalitis vectors) and anophelines using the in-house–developed Night Catcher, a modified CDC light trap that keeps caught adult mosquitoes fresh and alive for analysis and segregates hourly-caught mosquitoes in separate containers.

**Fig 8:**
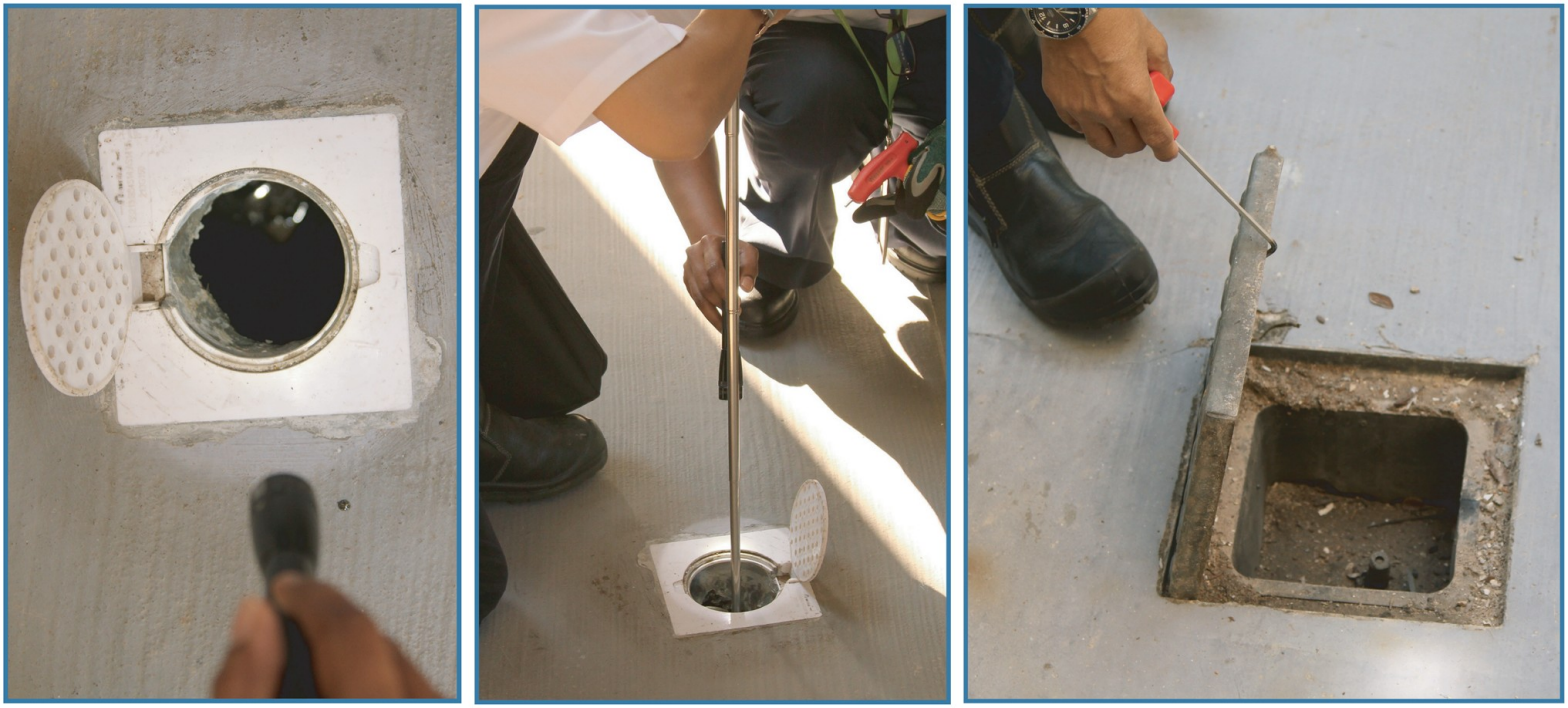
House inspections by NEA officers to identify *Aedes* habitats. NEA, National Environment Agency.

The Ministry of Health is responsible for case surveillance and clinical management of dengue patients. Dengue is a notifiable disease, meaning that medical practitioners and clinical laboratories must report clinically suspected and laboratory-confirmed cases. If a patient presents at a health facility with suspected dengue, then blood samples are taken for diagnostic testing at a hospital or private laboratories using either rapid diagnostic tests against non-structural protein 1 (NS1) and immunoglobulin M (IgM) antibodies or reverse transcriptase polymerase chain reaction (RT-PCR) for acute cases. Alternatively, samples can be sent to the EHI for diagnosis, and through this system, EHI is able to monitor circulating dengue serotypes [42]. An epidemiological investigation of each case, conducted by telephone or in person, is triggered by the Environmental Public Health Operations department at NEA, and case locations are plotted on a GIS interface. A dengue cluster is formed when two or more cases have onset within 14 days and are located within 150 m of each other (based on residential and workplace addresses, as well as movement history collecting during the epidemiological investigation). Dengue clusters are graded and the situation communicated to the public via the colour-coded Community Dengue Alert System: high-risk area 10 or more cases (red); high-risk area with fewer than 10 cases (yellow); and no new cases, under surveillance for the next 21 days (green) (Fig 8). Once a cluster is formed, this triggers the NEA Environmental Public Health Operations team to ramp up vector control activities in the cluster.

Entomological indices and dengue case numbers are not reliable measures for assessing the long-term impact of vector control programmes because of changes in surveillance and diagnostic capabilities over time [30]. Instead, NEA uses blood bank IgG seroprevalence to estimate the force of infection (FOI) over time [30].

### Scale-up and integration of vector control tools and approaches

The Singapore dengue programme relies predominantly on source reduction and larviciding using *Bti*, which are implemented throughout the year through community and programme efforts. In the first 6 months of 2019, approximately 60% of vector habitats were found in residential premises, rising to 70% in cluster areas. House inspections are conducted routinely and at increased frequency in cluster areas by the Environmental Public Health Operations team (Fig 8). Field staff follow a strict protocol including identifying themselves clearly and ensuring that the resident witnesses the taking of any samples for species identification at the EHI. Use of insecticides is seen as a short-term solution, so fogging with organophosphates is restricted to clusters during outbreaks only.

Source-reduction activities are facilitated by legislation and law enforcement, which Singapore uses in addition to community engagement to enhance public compliance. According to the Control of Vectors and Pesticides Act (CVPA, the main legislation dealing with mosquito breeding), the Operations team can enter homes to conduct inspections and vector control, and residents are fined at least S$200 (US$145) if aquatic stages of vectors are found on their premises [43].

Inspections of construction sites and other premises are also conducted by a dedicated NEA team. If aquatic stages of the vector are found, then an order can be served for vector control to be implemented within a specific time period by a registered vector control personnel. In the event that environmental management is found wanting, the CVPA allows NEA to issue an order to stop any work being undertaken on the premises indefinitely or until vector control to remove favourable habitats has been carried out. A list of construction sites that currently have Stop Work Orders are available on the NEA website (www.nea.gov.sg). Failure to comply with the order can lead to a fine of up to S$20,000 (US$14,500) and, in the case of a second or subsequent conviction, to a fine of up to S$50,000 (US$36,000) and/or imprisonment for up to 6 months. The CVPA also covers pesticides registration, as well as licensing and certification of private-sector vector control operators [43].

### Basic and applied research and innovation

To address challenges such as climate change, increasing urbanisation, and the manpower-intensive nature of source-reduction activities, the Singapore programme invests in research and development of new vector control technologies. For example, EHI is evaluating the use of *Wolbachia*, a maternally inherited endosymbiotic intracellular bacterium, which when artificially introduced into *A*. *aegypti* can suppress dengue infections. Singapore opted for a population-suppression strategy whereby only male *Wolbachia* mosquitoes are released. When male *Wolbachia* mosquitoes mate with wild-type females, the eggs produced will not hatch, because of a phenomenon called cytoplasmic incompatibility. Use of suppression was favoured over a population-replacement strategy, in which both males and females are released, since it is in line with the long-term programme strategy of source reduction. *Wolbachia* testing has followed a phased approach in two main release sites, including gaining an understanding of the basic biology of the released male *Wolbachia* mosquitoes in the field, such as how far and how high they fly. The programme has also collaborated with the International Atomic Energy Agency (IAEA) to irradiate *Wolbachia* mosquitoes at the pupal stage to render infertile any remaining females, so as to avoid replacement of the wild-type population with *Wolbachia A*. *aegypti* mosquitoes. Pilot testing has been accompanied by a careful community engagement campaign developed by EHI to emphasise that *Wolbachia* is safe and that male mosquitoes do not bite, and to encourage continued source reduction. The programme also works with private-sector collaborators to develop and evaluate technologies for *Wolbachia* testing and implementation. EHI works with Orinno Technology, a local start-up company, to develop new automated equipment to facilitate their work, including a larvae counting system, pupae sorting and counting system, and mosquito launcher to simplify and speed up the releases of the *Wolbachia* mosquitoes by field staff. The programme also works with Verily Life Sciences, an Alphabet, Inc., affiliate, to field-test the company’s automated sorting and release technologies.

The dengue control programme has developed a novel indicator for entomological risk assessment known as the *A*. *aegypti* breeding percentage [44]. Routine larval surveillance is not uniform spatially and temporally and so would be biased if used for risk assessment. Instead, the breeding percentage expresses the number of *A*. *aegypti*–positive habitats over the total number of *Aedes*-positive habitats (*A*. *aegypti* and *A*. *albopictus*) to cancel out the sampling error from nonsystematic inspection and cryptic breeding sites.

Predicting dengue outbreaks is difficult and the EHI has, in partnership with academic and research institutions including the National University of Singapore, developed several different risk models to enable better resource planning and preparedness for outbreaks. For example, a risk map is prepared each year to guide resource allocation to different areas [45], a temporal model is used to predict dengue cases up to 3 months in advance [46], and a spatiotemporal model integrating climate, vector density, population demographics (connectivity using public transport and mobile phone data), cases, infrastructure (age of building and number of units), and satellite data (vegetation) is used for high-resolution prediction and real-time allocation of resources [45].

### Building vector control capacity and capability

Staff personal and professional development is a focus of the NEA. For example, staff undertake continuing professional development courses organised by the internal training arm of NEA, the Singapore Environment Institute. Rotations between departments are encouraged and staff can receive financial support and leave of absence to attend further education. There are possibilities for both vertical and horizontal movement within the organisation, recognising the need for both specialists and generalists. The EHI partners with academic and research institutions, and staff members have obtained PhD and other degrees through their research work conducted at the EHI.

The EHI of NEA has been a WHO Collaborating Centre for Reference and Research of Arbovirus and their Associated Vectors since 2011. This involves consulting and advising (e.g., the Director of EHI sits on the WHO Strategic Technical Advisory Group on Neglected Tropical Diseases and Strategic Advisory Group of Experts Working Group for Dengvaxia, the Sanofi dengue vaccine), enhancing global outbreak preparedness (e.g., cross-border virus surveillance through UNITEDengue consortium, evaluation of diagnostics, etc.) and capacity building (e.g., training and sharing of best practice).

## Discussion

The Singapore dengue control programme is one of the best in the world, but what makes it so successful, and how can the lessons learnt be applied to other vector control programmes? Although in many countries dengue control sits under the Ministry of Health, unusually in Singapore it sits within the MEWR. This is in line with the programme view that dengue is an environmental disease. Dengue vector control uses mainly environmental management approaches, such as proactive source reduction, and environmental management including drainage and house improvements contributed to the elimination of malaria from Singapore in 1981 [34]. Key individuals including the Director-General of Public Health, could be trained as engineers or other professionals, not medical doctors. A strong sense of environmentalism stems from Singapore’s founding father, Lee Kuan Yew, who oversaw the transformation of Singapore after independence and in 1967 championed the idea of the ‘Garden City’ [47]. Lee Kuan Yew promoted a green environment that was free of litter in order to create good living conditions for Singapore’s residents, but also to simultaneously encourage tourism, investment, and trade. Strong political will and long-term political stability (the People’s Action Party have been the only party to form a government since independence in 1965) means that this vision and trajectory has been maintained over time, for example, in the current Sustainable Singapore Blueprint [15]. Despite sitting within MEWR, the programme collaborates closely with the Ministry of Health with efficient systems for sharing information on confirmed cases to allow rapid intervention by the NEA Environmental Public Health Operations team. Adopting a similar environmental approach to vector control could enable more effective control of VBDs worldwide (and incidentally was largely responsible for the success of vector control in the early and mid-1900s before the advent of DDT [6]). For example, progress in controlling malaria is stalling in many high-burden countries due to weak vector control programmes, and potentially also insecticide resistance [48, 49]. Since malaria is primarily a disease caused by standing water, proactively tackling immature vectors by using environmental management could be a synergistic addition to predominantly insecticide-based adult anopheline control.

Increasingly complicated and multidimensional public issues including climate change, globalisation, public health, and infectious disease outbreaks call for a transformation in public administration. Since the 1990s, Singapore has adopted a whole-of-government approach, which ‘denotes public service agencies working across portfolio boundaries to achieve a shared goal and an integrated response to particular issues’ [50–52]. The whole-of-government culture, propagated for decades in Singapore, facilitates the view of dengue control as a shared responsibility across agencies. For example, cross-sectoral collaboration for dengue control is facilitated by the Inter-Agency Dengue Taskforce. Further, rotation of leadership between different government departments and agencies means that so-called ‘T-shaped’ managers have not only specialist expertise but a broader perspective on issues and can help to break down departmental or agency silos [53]. This coordinated whole-of-government approach is exemplified by the Singapore response to a dengue outbreak that coincided with the start of the COVID-19 pandemic in early 2020 (Box 1).

**Box 1. Case study on intra- and intersectoral coordination to tackle dengue amid COVID-19**

Singapore’s dengue control response in 2020, which is taking place amid the COVID-19 pandemic, offers a case study of intra- and intersectoral collaboration to combat twin environmental public health threats.

**Detection of dengue threat and raising the alert**

More than 4,000 dengue cases were reported In the first quarter of 2020, double that for the same time period in 2019 [54]. In early 2020, EHI’s risk models, incorporating case data from the Ministry of Health, forecasted a dengue surge in the coming months. EHI’s analysis of serotype trends further detected an increase in the proportion of DENV-3 cases, with DENV-3 overtaking DENV-2 as the predominant serotype in early 2020. As DENV-3 has not been predominant in Singapore for nearly three decades, population immunity to this serotype is likely low, further increasing the risk of an outbreak. Given this outlook for 2020, NEA alerted relevant government agencies and set in motion an enhanced and coordinated dengue control response.

**Coordination of dengue control response**

With support from political and grassroots leaders, NEA brought forward the National Dengue Prevention Campaign (typically held just before the traditional midyear peak dengue season) to late March 2020 [54], with the intention of raising awareness and rallying the public to conduct preemptive measures early on. This kicked off island-wide community-led outreach efforts, helmed by grassroots leaders and supported by Dengue Prevention Volunteers, to encourage residents to carry out dengue prevention practices.

At the organisational level, the NEA-led Inter-Agency Dengue Taskforce met in January and March 2020 to coordinate the response across sectors and continues to meet regularly. Emphasis has been placed on enhancing vector control in assets managed by various agencies (such as buildings, reservoirs, drains, and parks), especially if these are located in areas with high mosquito populations or within dengue clusters.

**Dengue control in the time of COVID-19**

Singapore reported its first case of COVID-19 on 23 January 2020 [55]. To curb local COVID-19 spread, NEA in February 2020 launched “SG Clean” [56], a whole-of-government campaign to rally individuals and businesses to keep public areas clean (such areas include toilets, hawker centres, community spaces, and other premises). Dengue control messaging was woven into ‘SG Clean’. A key campaign message was that enhanced public cleanliness, such as maintaining clean premises and not littering, eliminates mosquito breeding habitats and hence helps to reduce the spread of dengue in addition to COVID-19.

In April 2020, Singapore implemented a ‘circuit breaker’ to curb COVID-19 transmission, which involved stringent social distancing measures and cessation of nonessential work activities [57]. Given the high-risk dengue outlook for 2020, NEA worked at the whole-of-government level to include vector control activities as an essential service to be continued during the ‘circuit breaker’ period. Businesses and owners of premises are expected to ensure that adequate vector control activities continue at their premises (including offices, commercial buildings, schools, and construction sites), even if regular operations are on hold. NEA continues to carry out island-wide routine inspections and enforcement, with precautions taken to minimise COVID-19 transmission. These precautions include ensuring that officers carrying out inspections are healthy, wear masks, and practise good personal and hand hygiene. Despite the efforts of the NEA, dengue control has been particularly challenging in 2020 because of the switch to DENV-3, warm weather, and high numbers of people staying at home during the ‘circuit breaker’ period, which can increase *Aedes* aquatic habitats and provides easy access to blood meals for female *Aedes* mosquitoes.

Singapore also employs collaborative governance, whereby governing is based on collaboration between government and nongovernment stakeholders. This is exemplified by the important role of the People’s Association in bridging between government and communities. Community outreach initiatives enjoy broad political support, as they provide an opportunity for the government to directly connect with the community. Local politicians are also invested in preventing outbreaks in their constituencies and often communicate the importance of dengue prevention to residents during walkabouts.

Another success factor may be the use of a ‘carrot and stick’ approach to source reduction with community engagement and behaviour change campaigns led by the 3P Network Division, backed up by strong legislation and enforcement. A recent study conducted by EHI shows that houses that have more frequent inspections have a lower number of reported mosquito larval habitats [58], lending support to the system of house inspections. A lack of corruption (Singapore is rated as one of the least corrupt nations in the world by Transparency International [59]) also supports the penalty system for vector habitats. The clear accountability of mosquito breeding offences under the law makes it easier for stakeholders to understand their respective roles, thus facilitating collaborative action against mosquito breeding. Strict enforcement of the law is another push factor that encourages joint efforts to take preventive measures. Imposition of fines for vector habitats may not be possible in all vector control programmes globally but should be considered.

What continues to drive transmission in Singapore, where there is a well-resourced and effective dengue control programme? The dengue incidence rate in Singapore has increased dramatically in the last 25 years, but this is likely because of improved diagnostics, increased referral for testing by medical practitioners, and increased awareness among the public [30]. A better indicator of the true infection rate is dengue FOI: between the 1960s (when Singapore first implemented environmental management and vector control programmes) and the 1990s, the dengue FOI in Singapore dropped 10-fold to approximately 0.01 (10 per 1,000 individuals per year) and has since held steady at this low level [30]. The decline in FOI can probably be attributed to the effectiveness of the dengue control programme (along with an increasingly ageing population). This success in reducing disease transmission has resulted in a lowered herd immunity against dengue, leaving Singapore’s population vulnerable to outbreaks despite a low vector population.

Another challenge is the high level of population movement in and out of the country, which is known to facilitate the co-circulation of DENV serotypes [60]. Singapore has 1,107 km of expressways and 199.3 km of mass rapid transit (MRT) lines across the island; over 300,000 people commute into the island state from Malaysia each day [61]; and in 2018, there were 65.63 million passenger movements in and out of Changi Airport from 380 cities in 100 countries and territories worldwide [62, 63].

In order to reduce the FOI further, the programme is evaluating innovative strategies such as *Wolbachia*, novel community engagement mechanisms, and risk mapping for more effective intervention targeting. As well as better implementation of current tools and approaches, new vector control tools are urgently needed to combat VBDs worldwide.

In conclusion, the dengue control programme in Singapore provides an excellent example of the GVCR in action. Important elements include strong collaboration across government departments and between government and nongovernment actors, integration of tools and approaches, effective surveillance, and community engagement. As a high-income country, Singapore is in an enviable position of having reliable government funding for dengue control. Nevertheless, aspects such as working across sectors or implementation of environmental management do not need to be expensive, and the former can even save costs for the vector control programme. Adoption of these elements could lead to more effective vector control programmes worldwide to reduce the intolerable burden of VBDs.

**Key Learning Points**

- VBDs are environmental diseases—situation of programmes within the Ministry of Environment and/or strengthening environmental management is encouraged.
- Whole-of-government approaches (working across government ministries) and collaborative governance (collaboration between government and nongovernment) support VBD control.
- Strong vector and epidemiological surveillance enables targeting of vector control interventions.
- Consider the role of legislation and enforcement in reducing vector habitats.
- Stable financing supports effective vector control.
- Innovation and science-based approaches should be harnessed to support surveillance and control.

**Top Five Papers**

1. World Health Organization. Global Vector Control Response 2017–2030. Geneva: WHO; 2017.
2. Tan LK, Low SL, Sun H, Shi Y, Liu L, Lam S, et al. Force of infection and true infection rate of dengue in Singapore: Implications for dengue control and management. Am J Epidemiol. 2019;188(8):1529–1538.
3. Shi Y, Liu X, Kok S-Y, Rajarethinam J, Liang S, Yap G, et al. Three-month realtime dengue forecast models: An early warning system for outbreak alerts and policy decision support in Singapore. Environ Health Perspect. 2015;124:1369–75.
4. Liang S, Hapuarachchi HC, Rajarethinam J, Koo C, Tang C-S, Chong C-S, et al. Construction sites as an important driver of dengue transmission: implications for disease control. BMC Infect Dis. 2018;18(1):382.
5. Aik J, Neo ZW, Rajarethinam J, Chio K, Lam WM, Ng L-C. The effectiveness of inspections on reported mosquito larval habitats in households: A case-control study. PLoS Negl Trop Dis. 2019;13(6):e0007492.
